# Analysis of Isotretinoin-Induced Alterations in the Levels of Plasma Trace Elements: Investigation of the Relationship Between Magnesium, Phosphorus, Potassium, Zinc, and Treatment-Related Side Effects

**DOI:** 10.1007/s12011-023-04053-9

**Published:** 2024-01-10

**Authors:** Tugrul Cagri Akman, Mustafa Yazici, Alptug Atila, Cuma Mertoglu

**Affiliations:** 1 Department of Analytical Chemistry, Faculty of Pharmacy, Erzincan Binali Yildirim University, 25240 Erzincan, Turkey; 2 Department of Dermatology, Faculty of Medicine, Erzincan Binali Yildirim University, 25240 Erzincan, Turkey; 3 Department of Analytical Chemistry, Faculty of Pharmacy, Ataturk University, 25240 Erzurum, Turkey; 4 Department of Clinical Biochemistry, Faculty of Medicine, Inonu University, 44280 Malatya, Turkey

## Abstract

Isotretinoin is an effective treatment against acne vulgaris, but it also causes many side effects during and after the treatment. The relationship between the changes in the levels of plasma trace elements of patients with AV after 3 months of isotretinoin treatment and the side effects was investigated in the study. Plasma samples of 35 patients were collected before and after isotretinoin treatment. Samples were analyzed by Inductively Coupled Plasma–Mass Spectrometer. After treatment, the levels of phosphorus, magnesium, and zinc in plasma increased statistically, while the level of potassium decreased (*p* < 0.05). The treatment had differing effects on zinc levels based on the gender of the individuals. With treatment, the levels of zinc in the plasma of men showed a greater increase compared to women (*p* = 7.3e-04). Additionally, the correlation matrix analysis revealed a strong correlation (*R* > 0.8) between magnesium and calcium. According to the study results, the change in phosphorus and potassium levels shows that isotretinoin affects kidney functions. The results suggest that phosphorus, potassium, magnesium, and zinc are associated with fatigue, dry skin and chapped lips, hair loss, and sebum secretion, respectively. Consequently, the study emphasizes the need for a comprehensive pre-treatment assessment, including monitoring of liver and kidney function as well as the levels of phosphorus and potassium in patients.

## Introduction

Acne vulgaris (AV) is a prevalent dermatological condition characterized by inflammatory eruptions in skin follicles. It is a multifactorial disease and its pathogenesis is still unclear. The intricate interplay of factors, encompassing sebum overproduction, heightened keratinization of pilosebaceous units, microbial colonization, and subsequent inflammatory responses, is proposed as the driving force behind the development of AV [1]. In light of these symptoms, therapeutic strategies are designed to match the disease’s severity, which includes a range of treatments that vary from topical agents and antibacterial therapies to oral antibiotics in milder cases. Systemic retinoid isotretinoin (ITR) has effective results, mainly when the treatments mentioned are insufficient. Furthermore, it is effective in treating chronic mild acne that rapidly resurges after the discontinuation of oral antibiotics, as well as moderate to severe AV [2].

Trace elements play a crucial role in various physiological processes and cellular mechanisms that occur at the cellular, chemical, and molecular levels. The concentration levels of trace elements in enzyme systems, blood, plasma, serum, and carrier proteins have a significant impact on human health and vary depending on several factors, including disease, treatment, and nutrition [3]. These changes in the concentrations can trigger alternative metabolic pathways and thereby potentially lead to various diseases and symptoms, because these effects on trace element homeostasis contribute to various symptoms and pathological conditions by altering liver enzymes, hematological profiles, and skin structure [4].

ITR is a synthetic version of vitamin A that is highly effective in treating AV. It can potentially provide long-term relief, and may even offer a permanent cure. However, its use is limited due to various side effects affecting the skin, mucosa, and other body parts. These side effects include cheilitis, dryness of the skin and mucosa, hair loss, nail fragility, diarrhea, abdominal pain, fatigue, and changes in liver enzymes and serum lipids [5–7]. During treatment, it is crucial to monitor the patient’s liver enzymes due to the teratogenic nature of ITR [8]. One significant factor contributing to the recurrence of AV is the discontinuation of ITR treatment. Significantly effective treatment and reducing acne recurrence rates without increasing side effects can be accomplished by utilizing higher doses of ITR. For this purpose, it is necessary to determine the risk factors associated with ITR that cause side effects. One of these risk factors is that the homeostasis of trace elements is affected by drug administration. Research on the levels of trace elements in AV patients has been widely reported. Only the correlation between AV and zinc was discussed in these studies [9–14]. However, there is only one study that specifically investigates zinc levels in AV patients using ITR [5]. There is no study available that investigates how ITR usage affects the plasma trace element levels in moderate-severity AV patients or establishes any relationship between these trace elements and the side effects of ITR.

The study centered on the assessment of biochemical parameters, side effects, and trace element concentrations in individuals with moderate-to-severe AV following a 3-month ITR treatment. The resulting findings will contribute to the assessment of treatment-related side effects in relation to alterations in trace element concentrations induced by ITR.

## Materials and Methods

### Reagents

Purified water, obtained using the Milli-Q Advanced A 10 purification system (Millipore, USA), was employed to prepare all aqueous solutions utilized throughout the study. For the preparation of plasma samples, internal standards, and standard solutions, a mixture solution comprising 1% HNO_3_ (Merck, USA) and 1% acetonitrile (Sigma Aldrich, Germany) was used. All equipment utilized in the study, including tubes, glass bottles, and micropipette tips, was cleaned with a 10% HNO_3_ solution, and washed with deionized water to avoid contamination during sample preparation and analysis. Agilent® Trace Elements solution contained the following standard trace elements at a concentration of 100 mg.L^−1^: lithium (Li), boron (B), sodium (Na), magnesium (Mg), aluminum (Al), phosphorus (P), sulphur (S), potassium (K), calcium (Ca), chromium (Cr), manganese (Mn), iron (Fe), cobalt (Co), nickel (Ni), copper (Cu), zinc (Zn), selenium (Se), strontium (Sr), cadmium (Cd), barium (Ba), lead (Pb) and bismuth (Bi). This solution was diluted to 1000 µg.dL^−1^ to establish the calibration curve for trace elements. The HNO_3_ used in the process was of 99.99% purity on a trace metal basis.

### Study Population and Collection of Plasma Samples

The investigation involved the inclusion of 35 patients, ranging in age from 18 to 40 years, who were in the process of seeking medical attention at the Dermatology Polyclinic situated within the Erzincan Mengücek Gazi Training and Research Hospital (Table 1). Plasma samples were collected from two different groups. The initial group, designated as “Pre-ITR” (*n* = 35), encompassed patients displaying moderate to severe AV without any prior exposure to oral ITR treatment. These patients exhibited an absence of any prior endocrine, renal, or hepatic abnormalities, as well as a lack of familial predisposition to the condition. ITR at a dose of 20 mg/day is indicated in cases of moderate to severe acne unresponsive to conventional treatment [15]. For this reason, the subsequent group, identified as “Post-ITR” (*n* = 35), was composed of the same individuals as in the “Pre-ITR” group, who took daily oral ITR treatment at a fixed 20 mg dosage for a 3-month ITR treatment. Individuals below 18 or above 40 years of age, those with mild facial acne vulgaris, any pre-existing kidney or liver diseases, prior hyperlipidemia, smoking or alcohol consumption, or individuals involved in dieting or strenuous physical activity were excluded from the study.

**Table 1:** Demographic characteristics of the patient group

Characteristic	Pre-ITR and Post-ITR (*n* = 35)
Male	16 (45.8%)
Female	19 (54.2%)
Age, mean ± SD, year	23.7 ± (4.82)
Age (range), year	18–40
Body Mass Index-BMI (kg/m^2^)	23.6
Disease duration, mean ± SD, year	2.6 ± 0.74
*Disease severity, n (%)* Moderate Severe	8 (22.85%) 27 (77.14%)

Patients participating in the study were instructed to maintain their regular diet and lifestyle throughout the 3-month ITR treatment. Blood samples of 5 mL were collected from all patients who visited the outpatient clinic and were diagnosed with AV, on an empty stomach, in the morning. Plasma was separated from these samples. After the 3-month ITR treatment, another set of 5 mL blood samples was obtained from the same patients during their follow-up visits to the outpatient clinic, again on an empty stomach, and plasma was separated from these samples as well. All plasma samples were stored at − 80 °C until trace element analysis following a series of biochemical tests.

Written informed consent was obtained from each patient. The study was carried out in accordance with the Declaration of Helsinki and approved by the Erzincan Binali Yıldırım University Clinical Research Ethics Committee on 26.04.2021 (Permission Number: E-21142744–804-0.99–77,978, Decision Number: 06/29). Biochemical tests were performed by the Dermatology clinic during the routine control of the patients. For ICP-MS analyses, plasma samples were removed from − 80 °C and kept at room temperature until thawed.

### Preparation of Plasma Samples for Inductively Coupled Plasma Mass Spectrometry (ICP-MS) Analysis

During the preparation of plasma samples for ICP-MS analysis, the Milestone Connect ETHOS UP microwave oven, and the Direct-Q 8 UV Ultrapure Water systems were used to prevent contamination from atmospheric particles. Firstly, the plasma samples were homogenized by vortexing for 1 min. Subsequently, 4.5 mL of ultrapure water was introduced to 0.5 mL of plasma sample, resulting in a 1:10 dilution, and the mixture was vortexed. Then, 0.5 mL of aliquots of the samples was transferred into teflon cups. 8 mL of HNO_3_ and 2 mL of H_2_O_2_ solutions were added to the mixture and subjected to microwave-induced degradation. The ramping conditions of the microwave program for the degradation process are given in Table 2.

**Table 2:** The ramping conditions of the microwave program

Step	Time	T1	T2	Pressure	Power
1	00:10:00	200 ℃	100 ℃	45 bar	Max power*
2	00:15:00	200 ℃	100 ℃	45 bar	Max power*

*Max power: 1500W for Ethos and 1200W for Start units

Following the degradation process, the volume of the samples was adjusted to 15 mL by adding ultrapure water. These samples were filtered with a 0.45 µm syringe tip. Finally, the samples were analyzed three times by ICP-MS, with the results averaged for accuracy. The dilution factor, as determined by the formula: “Dilution factor = (final weight or volume / sample quantity or volume) * dilution coefficient,” was employed to ensure the accuracy of the analytical process.

### ICP-MS Conditions

Data collection was carried out using the Agilent 7800 Quadrupole ICP-MS (Agilent Technologies, Japan), equipped with a rotary pump. This device featured an Integrated Sample Introduction System (ISIS 3) and Agilent ASX-500 Series ICP-MS Autosampler (Agilent Technologies, Japan) for sample handling. The Mass Hunter 4.2 Workstation Software 7800 ICP-MS Top C.01.02 was employed for device control and data processing. For quantitative mode ICP-MS analysis, a Ni sampler, MicroMist glass concentric nebulizer, and a quartz Scott type spray chamber were employed. Upon device activation, a sequence of essential tests was executed, encompassing Torch axis alignment, resolution axis calibration, EM calibration, standard lens tuning, plasma correction, full-spectrum analysis, and performance report assessments. Device calibration was executed utilizing the Agilent tune solution, which featured cerium, cobalt, lithium, magnesium, thallium, and yttrium at a concentration of 1 µg/mL. For trace element analysis, the helium collision mode was preferred, and argon gas was employed as the carrier gas. To maintain the analytical integrity of the ICP-MS system, a 45-min purification process with helium gas was conducted before the analysis. A comprehensive summary of the device’s settings and operating parameters, as applied in this methodology, can be found in Table 3. The autosampler, tubing, and probe were cleaned with 2% HNO_3_ and 1% HCl solutions, followed by ultrapure water, in preparation for sample injections.

**Table 3:** Agilent 7700 Quadrupole ICP-MS device parameters

Parameters	Value
Plasma conditions	Forward power 1200W
Plasma gas flow	15.0 L/min
Carrier gas flow	1 L/min
Carrier gas pressure	1.45 kPa
Dilution gas flow	1 L/min
He gas flow	4.5 mL/min
QP bias	− 15 V
Oct bias	− 18 V
Cell entrance	− 40 V
Cell exit	60 V
Deflect	− 0.8 V
Plate bias	− 60 V
Nebulizer pump speed	0.30 rps
Sample uptake rate	1.5 mL/min

### Method Validation

Before validation of the method and analysis of the plasma samples, routine quality control tests were carried out daily using Agilent-certified reference materials as per the manufacturer’s protocol for calibration and validation of the ICP-MS instrument. The data processing and measurement calculations were accomplished using the “Mass Hunter 4.2 Workstation Software 7800 ICP-MS Top C.01.02.”

The obtained results through linear regression analysis of the reference solutions were used to determine the calibration curve, curve equation, and correlation coefficient (*R*^2^). The method’s sensitivity was quantified by establishing the limit of detection (LOD) and the limit of quantification (LOQ) values. These LOD and LOQ values were determined as three and ten times the standard error of the curve slope, respectively (Table 4).

**Table 4:** Calibration curve equations, correlation coefficients and LOQ values of trace elements in the ICP-MS method

Trace elements	Calibration curve equations	Correlation coefficient	LOQ (µg.dL^−1^)
^7^Li	29.1 × + 16,356.2	0.9959	0.35
^11^B	3323.6 × + 30,541.6	0.9941	12.19
^23^Na	1,261,435.7 × + 469,916.3	1.0000	9.59
^24^ Mg	423,963.7 × + 9189.9	0.9999	3.92
^27^Al	67.2 × + 202.2	0.9950	0.13
^31^P	3063.3 × + 22,154.1	0.9992	23.21
^34^S	0.7 × + 2539.2	0.9960	173.14
^39^ K	326,304.0 × + 42,470.0	1.0000	0.13
^44^Ca	110,057.6 × + 410.0	0.9997	0.00
^52^Cr	5423.7 × + 1124.0	0.9947	0.21
^55^Mn	2098.4 × + 1030.0	0.9953	0.49
^56^Fe	4002.5 × + 20,570.2	0.9935	5.14
^59^Co	10,221.0 × + 333.3	0.9920	0.03
^60^Ni	2710.7 × + 7622.1	0.9943	2.81
^63^Cu	10,291.6 × + 10,010.7	0.9761	9.73
^66^Zn	1182.4 × + 3150.4	0.9968	26.64
^78^Se	58.8 × + 19.7	0.9993	3.35
^88^Sr	60,601.9 × + 5414.5	0.9952	0.09
^107^Ag	28,783.3 × + 1696.8	0.9950	0.06
^111^Cd	5574.6 × + 99.3	0.9948	0.02
^137^Ba	9204.8 × + 2146.9	0.9911	0.23
^208^Pb	30,599.1 × + 7619.0	0.9951	0.25
^209^Bi	46,524.7 × + 766.7	0.9956	0.02

*LOQ, limit of quantification

To assess the precision and accuracy of the ICP-MS method, intra-day and inter-day experiments, each involving three replicates of quality control (QC) samples, were conducted. The accuracy and precision of the method were thoroughly evaluated by calculating the percentage relative error (RE%) and percentage relative standard deviation (RSD%) values for both intra-day and inter-day analyses of the control samples. Accuracy was expressed through RE% values, while precision was quantified using RSD%. Acceptance criteria were established with values less than 20% deemed acceptable. Moreover, lead percent recovery values were considered satisfactory if they fell within the range of 80–120%.

### Statistical Analysis

The data obtained through ICP-MS analyses was visualized using RStudio (v. 1.3.1093). Trace elements were statistically analyzed using the Paired-Samples *t*-test (*p* < 0.05) with R program. The ANOVA analysis was conducted to determine if gender was a significant factor in trace element levels during ITR treatment. Normalization was not necessary as the variables had a normal distribution.

## Results

### Biochemical Tests

In the dermatology outpatient clinic, patients with ITR are monitored for their blood lipid levels and liver enzymes. At the start and after 3 months of ITR treatment, the clinic recorded the ALT (alanine aminotransferase), AST (aspartate aminotransferase), GGT (gamma-glutamyl transpeptidase) and ALP (alkaline phosphatase) liver enzymes of patients, as well as cholesterol, LDL, HDL, and triglyceride values from their blood samples. The data is analyzed using the Paired-Samples *t*-test, and the results are expressed as mean ± standard deviation. Table 5 shows the laboratory results of the 35 patients who participated in the study before and after 3 months of treatment.

**Table 5:** Alterations in the levels of blood lipids and liver enzymes following ITR treatment

Parameters	Pre-ITR	Post-ITR	Referance range	*p*-value
Cholesterol (mg.dL^−1^)	142.3 ± 3.7	210.4 ± 4.2	0–200	0.0246
HDL (mg.dL^−1^)	38.7 ± 1.2	58.3 ± 4.0	40–60	0.0676
LDL (mg.dL^−1^)	82.1 ± 1.6	111.6 ± 2.3	0–100	0.0374
Triglyceride (mg.dL^−1^)	70.7 ± 5.1	182.3 ± 8.7	0–200	0.0176
ALT (u.L^−1^)	9.30 ± 3.6	28.10 ± 7.6	7–35	0.0414
AST (u.L^−1^)	12.10 ± 0.4	26.10 ± 2.8	5–35	0.0220
GGT (u.L^−1^)	11.20 ± 1.7	18.20 ± 3.6	F: 0–45, M: 0–64	0.0131

*AST*, aspartate transaminase; *ALT*, alanine aminotransferase; *GGT*, gamma-glutamyl transferase; *LDL*, low density lipoprotein; *HDL*, high density lipoprotein, *p* < 0.05

After a 3-month treatment of oral ITR, participants experienced significant increases in their cholesterol, LDL, and triglyceride blood fat levels, as well as in their ALT, AST, and GGT liver enzyme levels (*p* < 0.05). Though there was also an increase in HDL, it was not statistically significant (*p* > 0.05). These changes are normal effects of the typical 3-month ITR treatment. Despite the increases in these parameters, only cholesterol levels remained slightly higher than the standard reference values as a result of the ITR treatment.

### Side Effects Observed After ITR Treatment

**Fig. 1:**
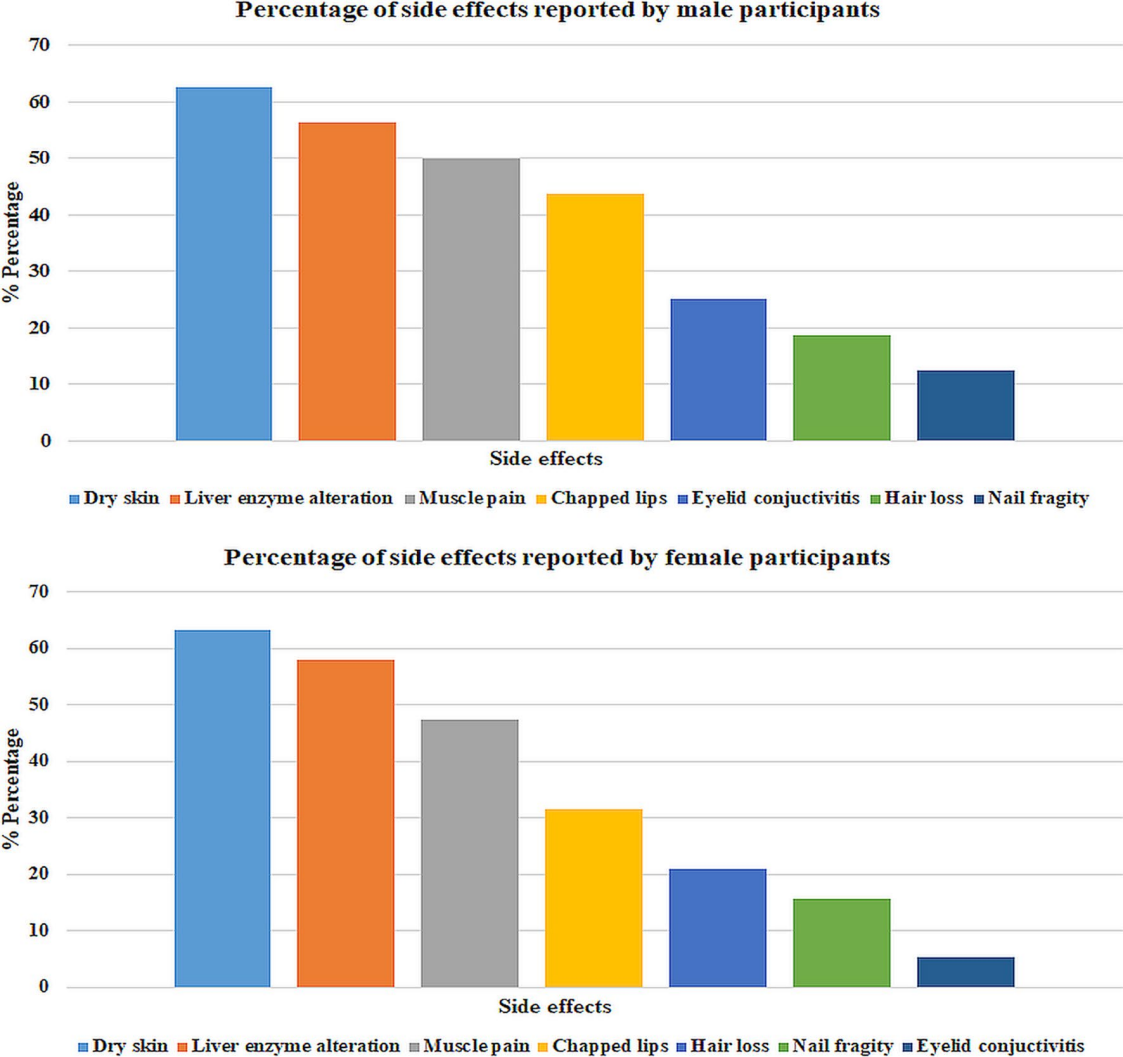
Percentage distribution of side effects reported by male and female patients with AV after ITR treatment

The side effects that most affected the patients after 3 months of ITR treatment in the study are presented in Fig. 1. Both male (62.5%) and female (63.16%) patients showed dry skin formation as the most frequently observed side effect. The least common side effect in male patients was nail fragility (12.5%), while in females it was eyelid conjuctivitis with 5.36%. All patients using ITR experienced varying severities of diarrhea, frequent urination, abdominal pain, and fatigue.

### Analyzing The Level of Trace Elements in Plasma Samples

The study used ICP-MS to measure the plasma levels of various elements including Li, B, Na, Mg, Al, P, S, K, Ca, Cr, Mn, Fe, Co, Ni, Cu, Zn, Se, Sr, Cd, Ba, Pb and Bi. After analysis, the study focused on 10 trace elements that were detected in over 80% of the plasma samples: Na, Mg, P, K, Ca, Cu, Zn, Se, Co, and Mn.

In the study, the concentrations of 22 trace elements (Li, B, Na, Mg, Al, P, S, K, Ca, Cr, Mn, Fe, Co, Ni, Cu, Zn, Se, Sr, Cd, Ba, Pb and Bi) in plasma samples were analyzed by ICP-MS. Meanwhile, the levels of Na, Mg, P, K, Ca, Cu, Zn, Se, Co, and Mn were accurately and precisely measured using the ICP-MS method. The plasma trace element levels of AV patients were subjected to statistical analysis through the Paired-Sample *t*-test using the R program, to assess the impact of ITR treatment. Table 6 indicates a significant effect of ITR treatment on trace element plasma levels with *p*-values less than 0.05.

**Table 6:** Changes of the trace element levels in plasma samples as a result of ITR treatment

Trace elements (µg.dL^−1^)	Pre-ITR (*n* = 35) Mean ± SD	Post-ITR (*n* = 35) Mean ± SD	*p*-value
Na	369.65 ± 3.01	371.89 ± 2.43	0.8785
Mg*	24.87 ± 0.83	41.45 ± 0.91	0.00049
P*	4250.3 ± 56.76	6337.2 ± 104.9	0.00023
K*	156.5 ± 2.87	115.7 ± 1.08	0.0010
Ca	3.3 ± 0.88	6.4 ± 0.76	0.2158
Cu	403.6 ± 4.07	732.8 ± 3.42	0.1849
Zn*	119.6 ± 6.49	207.5 ± 4.16	4.0e-04
Se (µg.L^−1^)	94.9 ± 0.08	102.2 ± 0.62	0.2728
Co	1.86 ± 0.25	1.97 ± 0.16	0.7631
Mn	2.47 ± 0.19	2.93 ± 0.68	0.6138

*p < 0.05

**Fig. 2:**
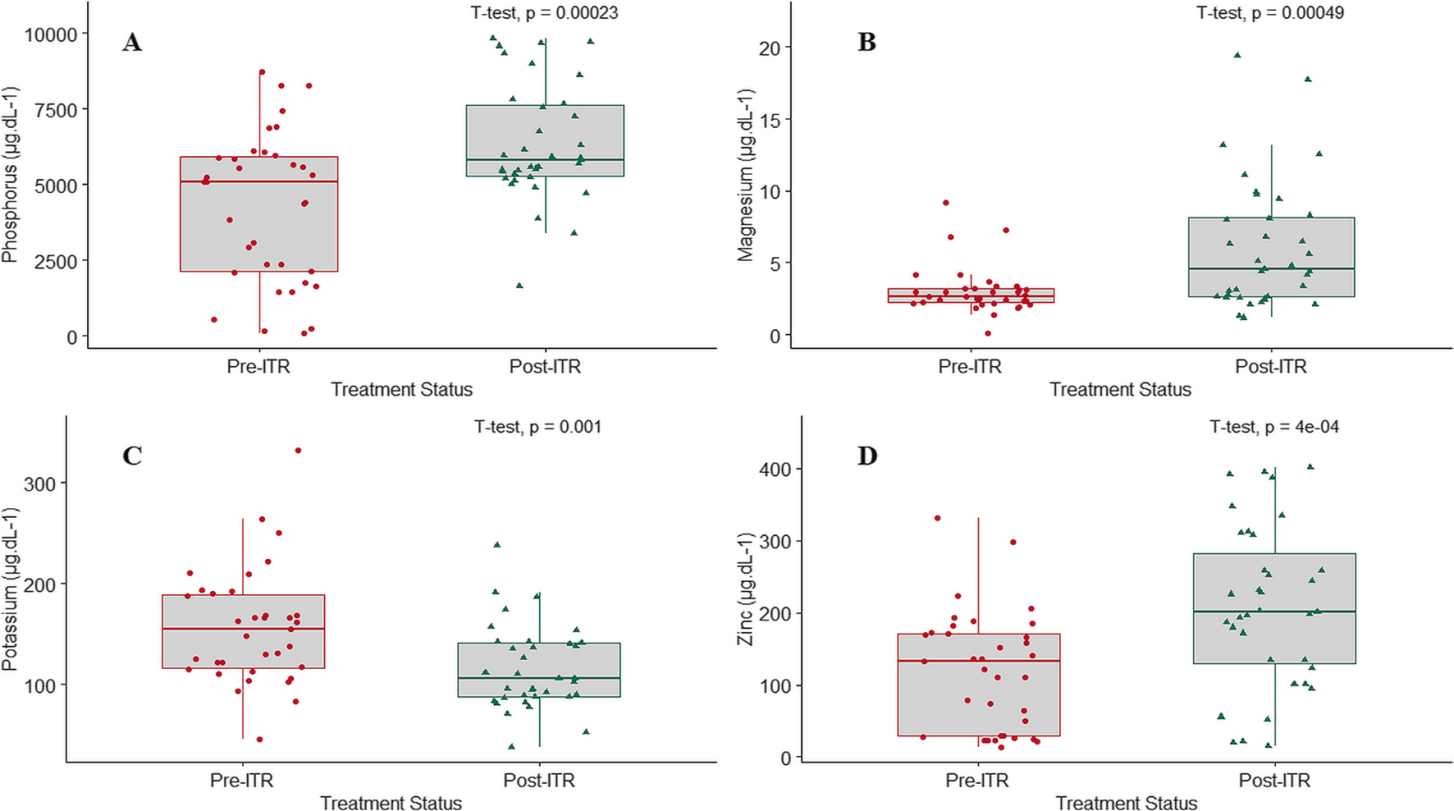
Boxplot graphs showing the effect of ITR treatment on plasma levels of (A) P, (B) Mg, (C) K and (D) Zn (*p* < 0.05)

There were statistically significant changes in the plasma levels of certain trace elements following ITR treatment. As seen in Fig. 2, the treatment increased plasma levels of P (*p* = 0.00023), Mg (*p* = 0.00049), and Zn (*p* = 4.0e-04), but decreased plasma levels of K (*p* = 0.0010). After systemic ITR treatment, plasma levels of Na, Ca, Cu, Se, Co, and Mn increased, but these increases were not statistically significant.

Cohen’s d effect size was used to determine the extent to which ITR treatment influenced the plasma concentrations of P, K, Mg, and Zn. According to the Cohen’s d criteria, the point estimate value of these trace elements being greater than 1.4 indicates that more than 92% of the difference in plasma concentrations of P, K, Mg, and Zn is due to ITR treatment [16]. Therefore, it can be concluded that ITR treatment has a significant impact on the plasma levels of these four trace elements in AV patients.

### Evaluation of Trace Element Changes After ITR Treatment According to Gender

Studying trace element levels before and after treatment based on gender is a common approach in medical and scientific research. This type of study can help researchers understand how various factors, including gender, may influence the levels of certain trace elements in the body and how they respond to treatment. For this aim, plasma trace element levels were assessed both before and after treatment, with a specific focus on analyzing potential gender-based differences in this study. According to one-way ANOVA analysis, the plasma K level (*F* = 7.42, *p* < 0.05) decreased significantly, while plasma Mg (*F* = 9.07, *p* < 0.05), P (*F* = 6.78, *p* < 0.05), and Zn (*F* = 10.12, *p* < 0.05) levels increased significantly after the treatment. Notably, both male and female participants experienced changes in the plasma concentrations of P, Mg, K, and Zn due to the ITR treatment (*p* < 0.05). For the AV patients, the plasma concentration of Zn in males was significantly higher than that of females both in the Pre- and Post-ITR groups (*t* = 5.49, *p* < 0.05; *t* = 3.27, *p* < 0.05) (Table 7).

**Table 7:** ANOVA analysis of trace element levels for evaluating gender-based differences

Trace elements	Gender	Pre-ITR	Post-ITR	*F*	*p-*value
Mg	Male (*n* = 16)	24.06 ± 0.1	40.83 ± 0.6	0.48	0.00053
	Female (*n* = 19)	25.61 ± 0.1	41.76 ± 1.3	1.72	0.00047
	Total	24.87 ± 0.8	41.45 ± 0.9	9.07	0.00049
	*t* for male vs. female	0.33	1.26		
	*p* for male vs. female	0.16	0.19		
P	Male (*n* = 16)	4168.1 ± 87.4	6164.4 ± 95.3	13.46	3.1e-05
	Female (*n* = 19)	4319.5 ± 94.7	6482.7 ± 124.9	8.37	2.64e-04
	Total	4250.3 ± 56.7	6337.2 ± 104.9	6.78	0.00023
	*t* for male vs. female	0.84	1.35		
	*p* for male vs. female	0.082	0.063		
K	Male (*n* = 16)	148.5 ± 3.6	113.4 ± 2.1	2.76	0.0009
	Female (*n* = 19)	163.5 ± 2.8	117.1 ± 0.9	1.17	0.0016
	Total	156.5 ± 2.8	115.7 ± 1.1	7.42	0.0010
	*t* for male vs. female	0.067	0.56		
	*p* for male vs. female	0.071	0.31		7262,5
Zn	Male (*n* = 16)	121.8 ± 8.1	211.1 ± 10.76	9.79	7.3e-04
	Female (*n* = 19)	117.4 ± 15.3	204.4 ± 9.72	11.68	2.1e-04
	Total	119.6 ± 6.49	207.5 ± 4.16	10.12	4.0e-04
	*t* for male vs. female	5.49	3.27		
	*p* for male vs. female	0.0096	0.017		

### Correlation Matrix

**Fig. 3:**
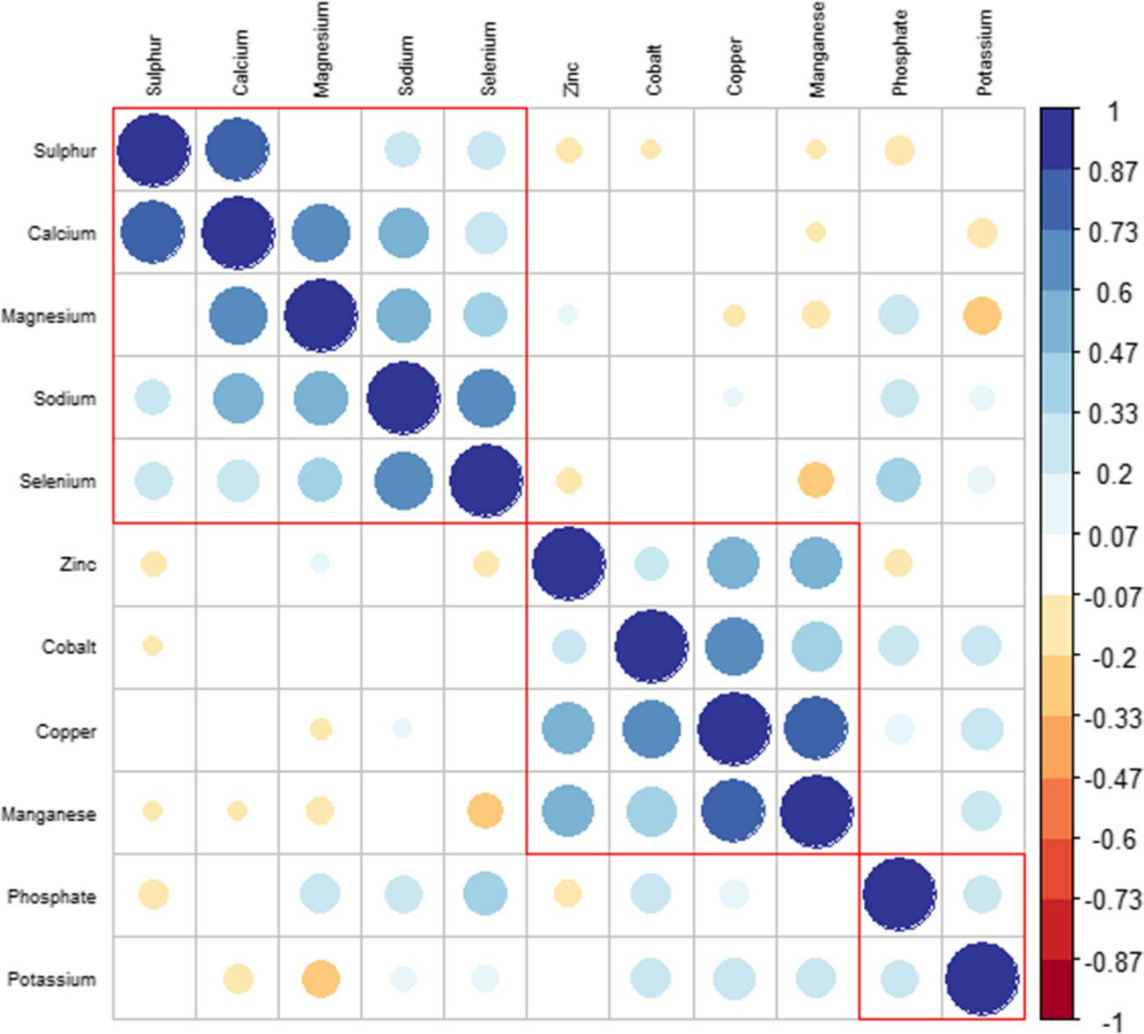
Spearman correlation matrix of plasma trace element levels. Positive and negative correlations are shown in blue and red, respectively

The ICP-MS results of plasma trace elements were analyzed using a correlation matrix. Eleven trace elements were measured in plasma samples, and it was found that their correlation coefficient with each other was higher than *R* > 0.8 (*R*, Spearman). This indicates that trace elements act together as a result of ITR treatment. The analysis also showed that there was a high correlation between Ca-S, Mg-Ca, Na-Se, Co–Cu, and Mn-Cu trace elements in plasma samples. Furthermore, it was observed that the plasma levels of Cu with Mn and Co, and Ca with S and Mg, increased after ITR treatment. Although there was no significant increase in Na and Se plasma levels, it was determined that there was a relationship between the two trace elements (Fig. 3).

## Discussion

Trace elements must be within certain concentration ranges to maintain biological, chemical, and cellular functions. Nonetheless, changes in levels of trace elements in plasma, serum, and bodily fluids — resulting from diet, drug therapy, or diseases — can adversely affect metabolism, organs, and tissues. Such alterations may cause negative effects on the body’s physiological processes, making it imperative to consider the importance of trace elements in patient care [17, 18]. Although various studies have examined the relationship between trace elements and AV, no studies have investigated the effect of ITR treatment on plasma trace element levels. The study focused on individuals receiving 3 months of ITR treatment to investigate the effect of this drug on plasma trace element levels and the potential relationship between these trace elements with a frequently reported side effects. The participants mostly reported experiencing side effects such as dry skin, conjunctivitis, chapped lips, hair loss, nail fragility, diarrhea, frequent urination, abdominal pain, and fatigue during ITR treatment in this study (Fig. 1). In the study, plasma samples were taken before and after treatment and the concentrations of 10 trace elements were measured using ICP-MS. According to statistical analysis, following a 3-month ITR treatment, there was a statistically significant increase in the concentrations of P, Mg, and Zn, while K concentrations exhibited a marked decline (*p* < 0.05, Table 6). Changes in P, Mg and K levels did not reveal any significant differences between male and female patients. However, male patients showed a significant increase in Zn levels after ITR treatment compared to females (*t* = 5.49, *p* < 0.05; *t* = 3.27, *p* < 0.05) (Table 7). On the other hand, the study showed that a strong correlation was found between some trace elements in the plasma samples at the end of ITR treatment, according to the correlation matrix analysis: Ca-S, Mg-Ca, Na-Se, Co–Cu, and Mn-Cu (Fig. 3).

Kidney function disorders can cause irregularities in the reabsorption and excretion of trace elements. Various forms of renal dysfunction, including acute interstitial nephritis, nephrotic syndrome, and hematuria with dysuria, have been reported to develop after 1 to 4 months of systemic oral ITR treatment at typical dosages (40 mg/day or 0.5 mg/kg/day) [19]. Another study reported that hematuria developed with the use of ITR. According to this study, after discontinuing isotretinoin, the presence of red blood cells in urine disappeared. Upon resuming isotretinoin, red blood cells reappeared in the urine [20]. Additionally, one of the renal dysfunctions, nephrotic syndrome, is known to increase plasma levels of P. Furthermore, hyperlipidemia that develops as a result of ITR treatment (Table 5) draws attention to nephrotic syndrome [21, 22]. Increased P levels due to ITR treatment in the study should suggest the development of nephrotic syndrome. Monitoring P levels during treatment may prevent the possible development of nephrotic syndrome. In addition, the routine urine analyses during treatment will help prevent the risk of hematuria before symptoms appear.

On the other hand, P is a critical element in numerous biological processes and is necessary for energy metabolism. It plays a crucial role in ATP (adenosine triphosphate) phosphorylation and cellular transport mechanisms [23]. Another significant result obtained in the study is that; both the increase in plasma levels and the fatigue symptoms observed in ITR users indicate that P cannot be used within the cell or is reduced [6, 7]. Enhanced energy demands due to various factors, including the pharmacological effects of ITR, could potentially lead to the depletion of intracellular P stores. This depletion may contribute to cellular fatigue, resulting in fatigue symptoms in patients using ITR. As a result, the increase in P levels indicated that this trace element was a potential contributing factor to the fatigue experienced by patients during ITR treatment.

The study brings to light significant differences, particularly in the regulation of K levels, attributable to the use of ITR in the AV treatment. The notable increase in urinary and fecal excretion, the side effects of ITR, can emerge as a prominent factor contributing to marked fluctuations in plasma K concentrations, both pre- and post-treatment. This study found an important reduction in plasma K levels in both male and female patients with AV after ITR treatment (Table 6 and 7). The authors think that this decrease is most likely due to diarrhea and frequent urination. K, which plays a role in cellular signaling and neuromuscular function, is also associated with fatigue and related symptoms [24]. The study highlighted the potential role of low plasma K levels contributing to fatigue and related symptoms during ITR use. The decline in plasma K levels could disrupt cellular signaling pathways and neuromuscular function, contributing to the fatigue and associated symptoms noted with ITR treatment. Furthermore, it is noteworthy that the decreased plasma K levels induced by ITR treatment may raise concerns regarding the potential development of nephrotic syndrome and dermatological issues, such as dry skin, chapped lips, and brittle nails, as reported in previous studies [22, 24].

These findings hold significant clinical implications, emphasizing the critical importance of meticulous pretreatment evaluations when considering ITR treatment for managing AV. Specifically, the study highlights the necessity of comprehensive assessments that encompass the measurement of patients’ baseline plasma K levels and renal function tests before initiating ITR treatment. Such an approach is advocated to preemptively address the potential development of K imbalances during the treatment, decreasing side effects and optimizing therapeutic outcomes.

Mg is an important cation found in high amounts in the human body for the execution of enzymatic functions and the regulation of various physiological processes. Maintaining Mg levels within the optimal range is crucial for overall health. Homeostasis of total body Mg levels relies predominantly on both gastrointestinal absorption and renal excretion and reabsorption [25]. In the study, plasma Mg concentration, which was measured as 24.87 ± 0.83 µg.dL^−1^ before treatment, showed a statistically significant increase as a result of ITR treatment (41.45 ± 0.91 µg.dL^−1^, p < 0.05) (Fig. 2B). This information and findings show that two different mechanisms may be involved in the increase in Mg levels. Firstly, the ITR treatment may have contributed to the reduction of renal excretion of Mg or increased its reabsorption within the renal tubules. This change in how the kidneys work led to an accumulation of Mg within the blood. Secondly, it is conceivable that ITR treatment might stimulate the release of Mg from bone stores, contributing to the observed increase in plasma Mg levels. However, the specific mechanisms governing these changes necessitate further investigation. Of particular interest is the potential link between elevated plasma Mg levels and changes in hair growth patterns, notably an increase in hair loss associated with ITR use. High levels of magnesium in the blood have a detrimental effect on hair growth and decrease the response to parathyroid hormone, a hormone that regulates Ca and Mg levels in the body. As a result, the hair cycle regulation can be influenced by the way Mg modulates the parathyroid hormone [25–27]. Consequently, there may be a link between increased hair loss and higher plasma Mg levels due to ITR use. The study provided important information to clarify the mechanism behind hair loss caused by ITR. Further research is needed to better understand the Mg homeostasis changes associated with ITR use.

Zn, an essential trace element, occupies a pivotal role in numerous critical biological functions, functioning as a fundamental constituent in various biomolecules and proteins. In healthy individuals, plasma Zn concentration is tightly regulated through homeostatic mechanisms, maintaining levels within a narrow range (70–150 µg.dL^-1^). This precise control of plasma Zn is facilitated by zinc ion-binding proteins, such as metallothioneins (MT), which, in response to external stimuli, can mobilize zinc from cellular compartments or stores into the circulatory system [28]. In recent years, the number of studies aiming to elucidate the relationship between serum Zn levels and AV were increased. However, the existing literature presents conflicting results. Notably, four studies have suggested significant differences in serum Zn levels between individuals with AV and healthy [11, 29–31]. Conversely, a controlled study encompassing a cohort of 200 patients failed to establish any substantial correlation between Zn levels and the occurrence or severity of AV [32]. Additionally, two more investigations revealed no significant association between serum Zn levels and AV [33, 34].

In contrast to these prevailing trends in the literature, this investigation deviates, revealing that individuals with AV exhibit elevated plasma Zn levels (averaging 119.6 ± 6.49 µg.dL^–1^). The data showed a statistically significant escalation in Zn concentration following a three-month ITR treatment (averaging 207.5 ± 4.16 µg.dL^–1^, *p* = 4.0e-04). This observed rise in plasma Zn levels can reasonably be attributed to the stimulation of MTs by ITR, thereby promoting the release of Zn into the bloodstream. There are only several studies supporting the effectiveness of oral Zn supplementation in reducing AV severity and sebum secretion [29, 35]. In light of these supportive findings and the distinctive outcomes of this study, it is plausible to suggest that ITR reduces AV symptoms by increasing plasma Zn levels through the stimulation of MTs. As such, this study offers a new perspective on the role of Zn and ITR in AV pathophysiology, offering valuable information on the pharmacological mechanism of ITR.

## Conclusions

In conclusion, the outcomes of this investigation shed light on the significant impact of ITR treatment on the homeostasis of trace elements, offering insights into the potential relationship between the observed symptoms (diarrhea, dry skin, hair loss, fatigue) in patients utilizing ITR and the levels of trace elements. Moreover, the study provided significant insights for future studies aiming to optimize ITR treatment for AV. The findings highlighted suggest the possibility of a wider pretreatment assessment with comprehensive biochemical analyses including analysis of kidney function and the levels of P and K to avoid unnecessary side effects. The study revealed that urine tests should be monitored regularly to prevent the risk of hematuria caused by ITR. In addition, the treatment had a differential impact on Zn levels depending on the gender of the individuals involved. This approach aims to enhance the safety and effectiveness of ITR treatment while mitigating the potential for associated side effects and kidney function disorders.

## Data Availability

Data sharing is applicable.
